# Supplementary material for: Renal Endocytic Regulation of Vitamin D Metabolism during Maturation and Aging in Laying Hens
Source: Animals (Basel). 2024 Feb 2;14(3):502. doi: 10.3390/ani14030502 (PMC10854989; doi:10.3390/ani14030502)
Supplement: Supplementary file 1 [file animals-14-00502-s001.zip › animals-2849533-supplementary.pdf]

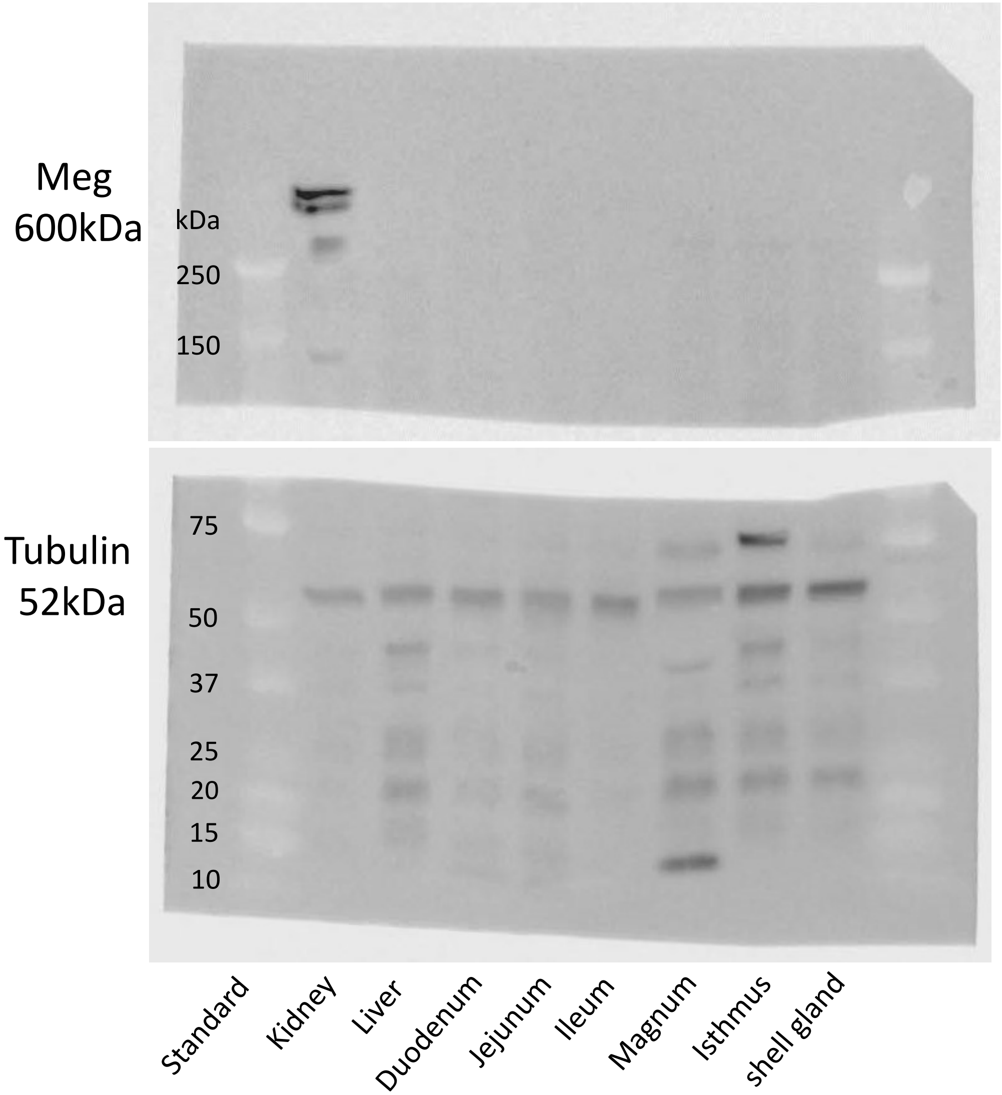

The Original PVDF membranes of western blotting analysis for megalin (Meg) and tubulin (Tubulin) proteins (manuscript Figure 3A). PVDF membrane was cut at approximately 100 kDa; the upper membrane was used for Meg chemiluminescence detection, and the lower membrane was used for tubulin chemiluminescence detection. White incident light figures of Precision Plus Protein Dual Color standard (Bio-Rad) were overlaid on the Meg and tubulin figures.

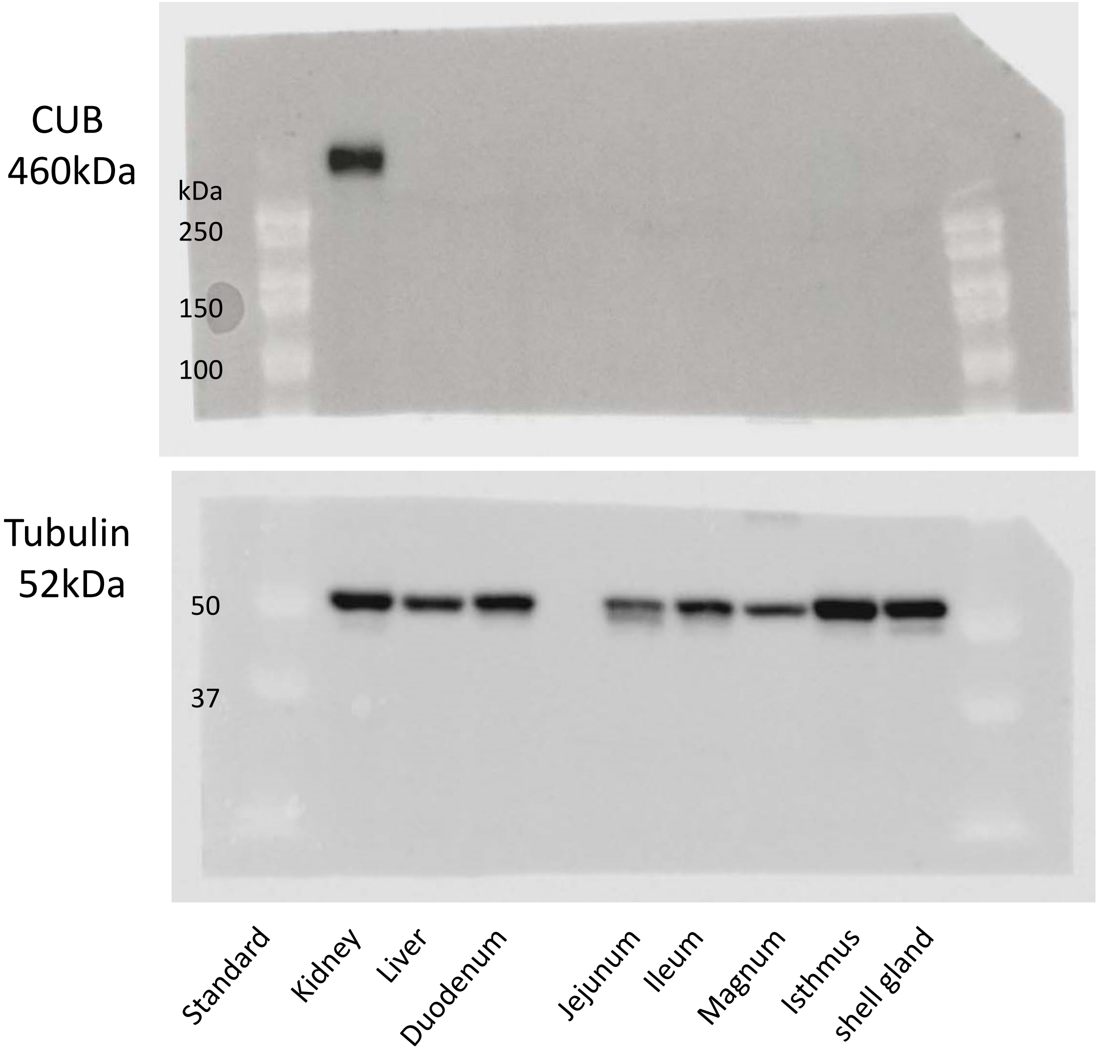

The Original PVDF membranes of western blotting analysis for cubilin (CUB) and tubulin (Tubulin) proteins (manuscript Figure 3B). PVDF membrane was cut at approximately 75 kDa; the upper membrane was used for CUB chemiluminescence detection, and the lower membrane was used for tubulin chemiluminescence detection. White incident light figures of Precision Plus Protein Dual Color standard (Bio-Rad) were overlaid on the CUB and tubulin figures.
